# Transcriptomic and Metabolomics Joint Analyses Reveal the Influence of Gene and Metabolite Expression in Blood on the Lactation Performance of Dual-Purpose Cattle (*Bos taurus*)

**DOI:** 10.3390/ijms252212375

**Published:** 2024-11-18

**Authors:** Shengchao Ma, Dan Wang, Menghua Zhang, Lei Xu, Xuefeng Fu, Tao Zhang, Mengjie Yan, Xixia Huang

**Affiliations:** 1 College of Animal Science, Xinjiang Agricultural University, Urumqi 830099, China; shengchaomasicau@163.com (S.M.); wangdan01100330@163.com (D.W.); zhangmenghua810@126.com (M.Z.); q609468041@sina.com (L.X.); z13319734240@163.com (T.Z.); y13095066028@163.com (M.Y.); 2 Institute of Animal Science, Xinjiang Academy of Animal Sciences, Urumqi 830011, China; fuxuefeng@xjaas.net

**Keywords:** dual-purpose cattle, blood, lactation performance, metabolomics, transcriptomics, joint analysis

## Abstract

Blood is an important component for maintaining animal lives and synthesizing sugars, lipids, and proteins in organs. Revealing the relationship between genes and metabolite expression and milk somatic cell count (SCC), milk fat percentage, milk protein percentage, and lactose percentage in blood is helpful for understanding the molecular regulation mechanism of milk formation. Therefore, we separated the buffy coat and plasma from the blood of Xinjiang Brown cattle (XJBC) and Chinese Simmental cattle (CSC), which exhibit high and low SCC/milk fat percentage/milk protein percentage/lactose percentages, respectively. The expression of genes in blood and the metabolites in plasma was detected via RNA-Seq and LC-MS/MS, respectively. Based on the weighted gene coexpression network analysis (WGCNA) and functional enrichment analysis of differentially expressed genes (DEGs), we further found that the expression of genes in the blood mainly affected the SCC and milk fat percentage. Immune or inflammatory-response-related pathways were involved in the regulation of SCC, milk fat percentage, milk protein percentage, and lactose percentage. The joint analysis of the metabolome and transcriptome further indicated that, in blood, the metabolism pathways of purine, glutathione, glycerophospholipid, glycine, arginine, and proline are also associated with SCC, while lipid metabolism and amino-acid-related metabolism pathways are associated with milk fat percentage and milk protein percentage, respectively. Finally, related SCC, milk fat percentage, and milk protein percentage DEGs and DEMs were mainly identified in the blood.

## 1. Introduction

Blood is a red, opaque, thick fluid that flows through an animal’s blood vessels, and it is a mixture of plasma and various immune cells, such as lymphocytes, neutrophils, and monocytes [1,2]. Many hormones, nutrients, and other information substances can reach their target organs through blood transport and coordinate the growth, development, and function of the entire body [1,2].

The production of milk is a complex process. After their offspring are born, mammals produce milk by reducing estrogen and prolactin levels in the body and increasing the secretion of prolactin to the mammary epithelial cells such that enzymes related to milk production are phosphorylated and activated to promote the synthesis of milk fat, milk protein, and lactose. During this process, prolactin is secreted by the pituitary gland and acts on the breast tissue through blood circulation. Inorganic salts, minerals, antibodies, and substances required for the synthesis of proteins (or milk fat and lactose) in milk need to be transported to the breast tissue through blood [3,4,5,6]. In addition, immune cells in the blood play a key role in the onset, progression, and regression of bovine mastitis. During infection with mastitis, they are substantially attracted to breast tissue and transferred into milk [7]. To summarize, the influence of blood on milk performance cannot be ignored; it can accurately reflect the physiological state of cows during lactation.

Compared with bovine mammary epithelial cells, blood is an easily obtained and minimally destructive tissue sample [8]. The gene expression profile extracted from blood provides a new opportunity to elucidate the molecular regulatory mechanism of bovine lactation performance, and biomarkers in blood can play an important role in describing the incidence status of mastitis [8,9,10]. Until now, relevant studies have mainly focused on screening candidate genes related to bovine mastitis resistance from blood. Wang et al. compared the blood transcriptomes of healthy and mastitis XJBC and discovered that *RHO*, *RCVRN*, *CSF1 R*, *CAV3*, *GATA4*, and other genes were linked with XJBC mastitis resistance [11]. Zhong et al. discovered that *FHIT* and *PIAS1* genes were linked to XJBC mastitis in blood using pyrosequencing and RT-qPCR [12]. Yang et al. characterized the peripheral blood transcriptomes of healthy and subclinical mastitis cows and used RNA-seq to search for the regulatory characteristics of subclinical mastitis in cattle. They found that three lncRNAs may affect the incidence of mastitis in dairy cows by upregulating the expression of *TLR4*, *NOD2*, *CXCL8*, and *OAS2* genes [8]. Luoreng et al. used RNA-Seq to construct miRNA expression profiles in the buffy coat of the blood of cows at different times after infection and further found that three miRNAs might be involved in the immune process during the late stage of *E. coli*-induced mastitis [13]. However, due to the complexity of this mechanism, the results obtained from these studies are different, and the current understanding of the molecular regulatory mechanism of mastitis resistance is still not comprehensive. In addition, some studies have shown that small molecule metabolites in blood may also affect the milk SCC, and changes in the metabolic pathways in blood correspond to the inflammatory damage of mammary tissue [14,15,16]. Several serum metabolites were used to distinguish subclinical mastitis cows from healthy cows up to 8 weeks before their due date using targeted GC-MS [15]. A recent study by Haxhiaj et al. confirmed that lactic acid in the blood is a biomarker of a high SCC at the 4-week prenatal period and at conventional diagnosis [17].

With the development of science, a large number of omics sequencing technologies continue to emerge and spread, producing a large number of omics data, including those related to the genome [18], transcriptome [19], metabolome [20], etc. These data provide a basis for further studies and the disclosure of key candidate genes and marker metabolites that affect milk performance. The joint analysis of the metabolome and transcriptome can supplement missing or unreliable information in the field of omics, and richer and more comprehensive information can be obtained by exploring the common pathways and differential expression of genetic material at different levels and the overall dynamic change law at the system level [21,22].

XJBC and CSC are the two largest dual-purpose cattle breeds in Xinjiang, China. They have the following characteristics: high stress resistance and resistance to rough feeding. Improving their lactation performance is crucial for improving the economic income of herdsmen and developing the local dairy industry. Therefore, this study uses XJBC and CSC as the research objects. Based on the indicators of dairy herd improvement (DHI is a standard index for evaluating the milk production performance of dairy cows, including the SCC, milk fat percentage, milk protein percentage, and lactose percentage), XJBC and CSC cattle with high or low SCC (or milk fat percentage, milk protein percentage, and lactose percentage) were selected for blood collection. The expression of genes in blood and metabolites in plasma was detected using RNA-Seq and LC-MS/MS techniques, respectively. Subsequently, based on bioinformatics and multivariate statistical analyses, DEGs and DEMs related to lactation performance were screened relative to the transcriptome and metabolome, respectively, to explore the relationship between DEGs and DEMs. Finally, the key candidate genes, marker metabolites, and molecular signaling pathways affecting the milk performance of dual-purpose cattle were revealed. The findings of this study provide a theoretical foundation for improving milk performance and investigating molecular control mechanisms during milk production.

## 2. Results

### 2.1. PCA and WGCNA of XJBC and CSC Blood Transcriptome Data

**Figure 1:**
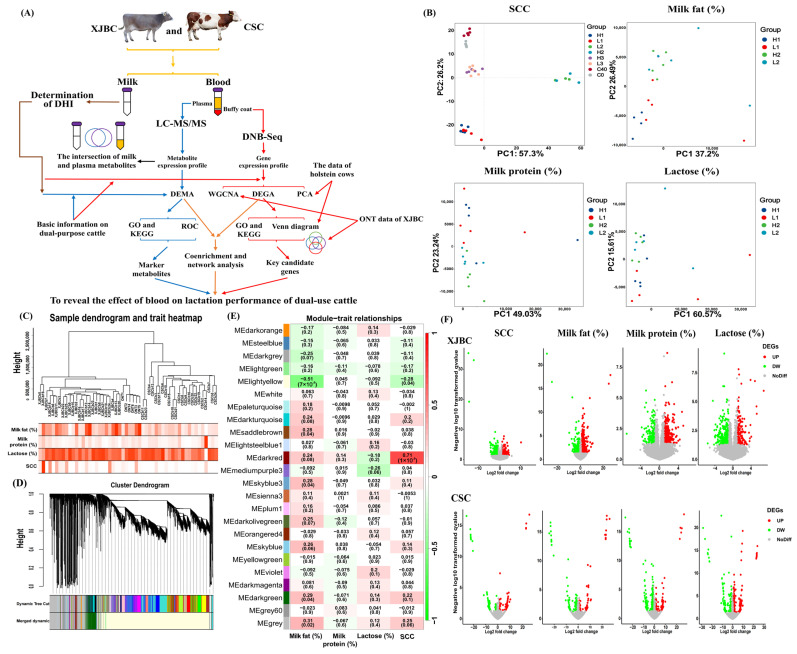
Transcriptomics reveals the association between gene expression in blood and milk DHI indicators. (**A**) The workflow of this study. DEMA: DEM analysis; DEGA: DEG analysis. (**B**) PCA results of transcriptome samples. H1: High−phenotype−value individual (XJBC); L1: low−phenotype−value individual (XJBC); H2: high−phenotype−value individual (CSC); L2: low−phenotype−value individual (CSC). C0: control (Holstein); C40: 40 days after infection with bacteria (Holstein). Transcriptome data (GSE159286) for Holstein cows were downloaded from the GEO database and uploaded by Cebron et al. [23]. (**C**) Cluster trees of transcriptome samples. (**D**) Clustering dendrogram of genes, with dissimilarity based on topological overlap, together with assigned, merged module colors and the original module colors. (**E**) Heat map of the correlation between modules and phenotypes. The correlation coefficient (significance *p*−value) was shown in the box. (**F**) Volcanic map of DEGs. Number (*n*) of samples in each group of XJBC or CSC: low or high SCC group: *n* = 5. Low or high milk fat percentage group: *n* = 5. Low or high milk protein percentage group: *n* = 5. Low or high lactose percentage group: *n* = 5.

First, the workflow of this study is shown in Figure 1A. PCA and sample cluster analysis showed (Figure 1B,C) that XJBC and CSC samples in the SCC, milk fat percentage, lactose percentage, and milk protein percentage groups were significantly distinguished, but the distinction between samples with high and low SCC, milk fat percentage, lactose percentage, and milk protein percentage was not obvious. The high and low SCC (or milk fat percentage) XJBC samples were separable only from the PC2 perspective. Based on the expression pattern, WGCNA further divided the whole genome genes into 24 modules, among which the lightyellow (containing 11,789 genes), saddlebrown (containing 69 genes), skyblue3 (containing 49 genes), darkgreen (containing 1164 genes), and grey (containing 3209 genes) modules were closely related to the milk fat percentage (*p* < 0.05). The lightyellow and darkred modules (containing 121 genes) were closely related to SCC (*p* < 0.05) (Figure 1D,E). In addition, we also noted that, in XJBC, SCC was significantly correlated with milk fat percentage (positive correlation), milk protein percentage (positive correlation), and lactose percentage (negative correlation) (*p* < 0.05) (Table 1). In CSC, SCC was also significantly correlated with milk protein percentage (positive correlation) and lactose percentage (negative correlation) (*p* < 0.05), but there was no significant negative correlation between SCC and milk fat percentage (*p* > 0.05) (Table S1).

GO and KEGG enrichment analyses showed that the genes in the lightyellow module were related to transcriptional regulation, protein phosphorylation and ubiquitination, inflammatory or immune response signaling pathways, cell cycle and apoptosis, the chemokine signaling pathway, amino acid metabolism, carbohydrate metabolism, fatty acid or phospholipid metabolism, purine and pyrimidine metabolism, etc. The genes in the darkred module were related to immune responses, bacterial defense responses, lipopolysaccharide binding, etc. Simultaneously, the genes in the saddlebrown module were related to mitochondria, ribosomes, translation, etc. The genes in the skyblue3 module were related to membrane and transmembrane transport. The genes in the darkgreen module were related to the development of the nervous system and signal transduction, bacterial defense response, lipid biosynthesis, lipid oxidation, the regulation of lipolysis in fat cells, cholesterol metabolism, etc. Finally, in the grey module, genes were related to nervous system development and signal transduction, bile acid and bile salt transport, immune-response-related signal pathways, linolenic acid metabolism, arachidonic acid secretion and metabolism, the negative regulation of fatty acid oxidation, fat digestion and absorption, the regulation of lipolysis in adipose cells, linoleic acid metabolism, ABC transporter, etc. (Supplementary File S1).

### 2.2. DEG Analysis

DEG analysis showed that in XJBC, 497 DEGs (260 were downregulated and 237 were upregulated; *FOS*, *IL10*, *GRO1*, *CCL3*, *ICAM1*, *VCAM1*, and other genes were located in the core node of the PPI network) were screened in low SCC vs. high SCC samples. In total, 968 DEGs (348 were downregulated, and 620 were upregulated; *FOS*, *MMP9*, *CCL2*, *KDM6 B*, *PTGS2*, *IL6*, *THY1*, *IL2*, *CXCR1*, *IL10 RA*, and other genes were located in the core node of the PPI network) were screened with respect to low milk fat vs. high milk fat percentages. Moreover, 503 DEGs (178 were downregulated, and 325 were upregulated; *IL1 B*, *MMP9*, *PTGS2*, *IL6*, *TNFAIP6*, *SERPINB2*, *LCN2*, and other genes were located in the core node of the PPI network) were screened relative to low milk protein vs. high milk protein percentages. Finally, 462 DEGs (295 were downregulated and 167 were upregulated; *PTGS2*, *IL2*, *CSF3*, *PPBP*, *CXCL5*, *CXCR1*, *CCL4*, *CXCR2*, *CCR4*, *CXCL3*, and other genes were located in the core node of the PPI network) were screened relative to low lactose vs. high lactose percentages (Figure 1F and Figure S2, Supplementary Files S2 and S6).

In CSC, 279 DEGs (155 were downregulated and 124 were upregulated) were screened in the low SCC vs. high SCC samples. In total, 318 DEGs (180 were downregulated, and 138 were upregulated; *MMP9*, *PXDN*, *PTI*, *CCNB1*, *CDC20*, *CDCA3*, and other genes were located in the core node of the PPI network) were screened relative to the low milk fat vs. high milk fat percentages. Then, 519 DEGs (155 were downregulated, and 159 were upregulated; *GNG4, GNAI1, CCL27, CX3 CR1*, and other genes were located in the core nodes of the PPI network) were screened relative to low milk protein vs. high milk protein percentages. In total, 554 DEGs (155 were downregulated, and 124 were upregulated; *IL1 A*, *CCL3*, *MMP9*, *GRO1*, *CXCR2*, *CXCL3*, *CXCL2*, *TNF*, *CCL4*, *PPBP*, *BCL6*, and other genes were located in the core node of the PPI network) were screened relative to low lactose vs. high lactose percentages (Figure 1F and Figure S2, Supplementary Files S2 and S6). GO and KEGG enrichment analyses showed that in XJBC and CSC, more DEGs related to the SCC, milk fat percentage, milk protein percentage, and lactose percentage (including most of the DEGs located at the core node of the PPI network) were significantly enriched relative to the immune and inflammatory response pathways (Supplementary File S3).

**Figure 2:**
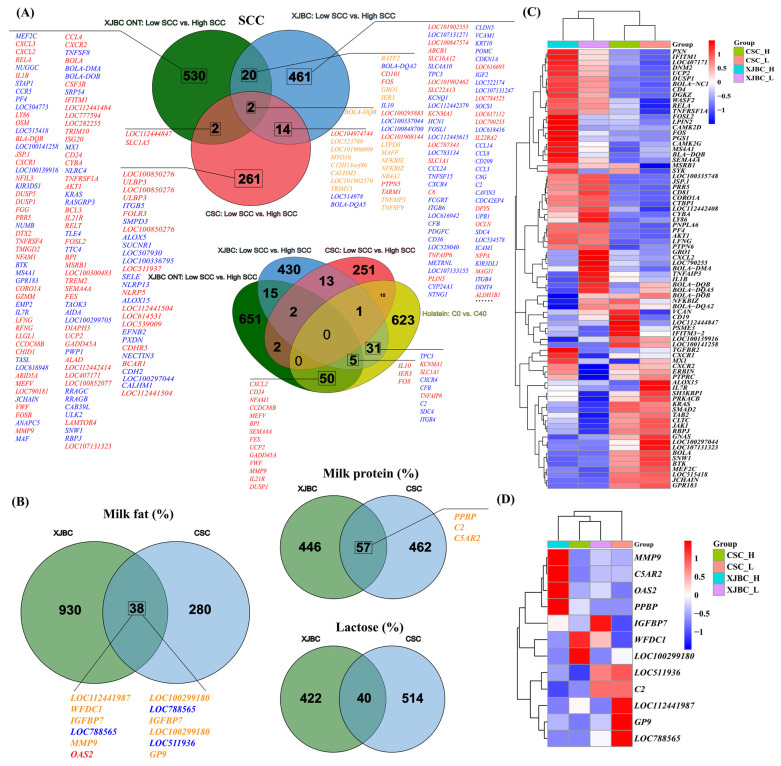
Common DEGs of XJBC and CSC. (**A**) DEGs intersection of XJBC ONT: low SCC vs. high SCC; XJBC: low SCC vs. high SCC; CSC: low SCC vs. high SCC; Holstein: C0 vs. C40. The blue and red genes in this figure are DEGs with downregulated or upregulated expressions in different comparison groups, respectively, while the expression trend in orange DEGs is opposite in different comparison groups. (**B**) DEG intersection of XJBC: low milk fat percentage (or milk protein percentage and lactose percentage) vs. high milk fat percentage (or milk protein percentage and lactose percentage) and CSC: high milk fat percentage (or milk protein percentage and lactose percentage) vs. low milk fat percentage (or milk protein percentage and lactose percentage). (**C**,**D**) expression patterns of common DEGs located in the signaling pathways associated with immune or inflammatory responses.

Based on the ONT sequencing data of XJBC blood with high and low SCC, we further lowered the DEG screening threshold from Yan et al.’s study [24], reperformed the DEG analysis, and finally, obtained 725 DEGs, which were included in the Venn diagram analysis. The Venn diagram analysis showed that (Figure 2A) the intersection of the three comparison groups contained 2 DEGs; the intersection of XJBC ONT: low SCC vs. high SCC and XJBC: low SCC vs. high SCC contained 22 DEGs; the intersection of XJBC: low SCC vs. high SCC and CSC: low SCC vs. high SCC contained 16 DEGs; the intersection of XJBC ONT: low SCC vs. high SCC and CSC: low SCC vs. high SCC contained 4 DEGs. Among these common DEGs, *BOLA-DQB*, *BOLA-DQA2*, *FOS*, *GRO1*, *IER3*, *IL10*, *LOC100848700*, *NFKBIE*, *NFKBIZ*, *NR4 A1*, *TNFAIP3*, *TNFSF9*, *LOC100337108*, *LOC100337076*, *ALPL*, *LOC514978*, and *BOLA-DQA5* genes were enriched in pathways associated with immune and inflammatory responses (Supplementary File S3): immune response; inflammatory response; antigen processing and the presentation of peptide or polysaccharide antigens via MHC class II; immunoglobulin production relative to immunoglobulin-mediated immune response; peptide antigen assembly with an MHC class II protein complex; peptide antigen binding; MHC class II protein complex binding; Th1 and Th2 cell differentiation; Th17 cell differentiation; TNF signaling pathway; and NF-kappa B signaling pathway. Furthermore, the intersection of DEGs associated with milk fat percentage, milk protein percentage, and lactose percentage in CSC and XJBC contained 38, 57, and 40 DEGs, respectively (Figure 2B). Figure 2C,D show the expression patterns of common DEGs associated with inflammation and immune response.

### 2.3. Review of Plasma Metabolites of XJBC and CSC

**Figure 3:**
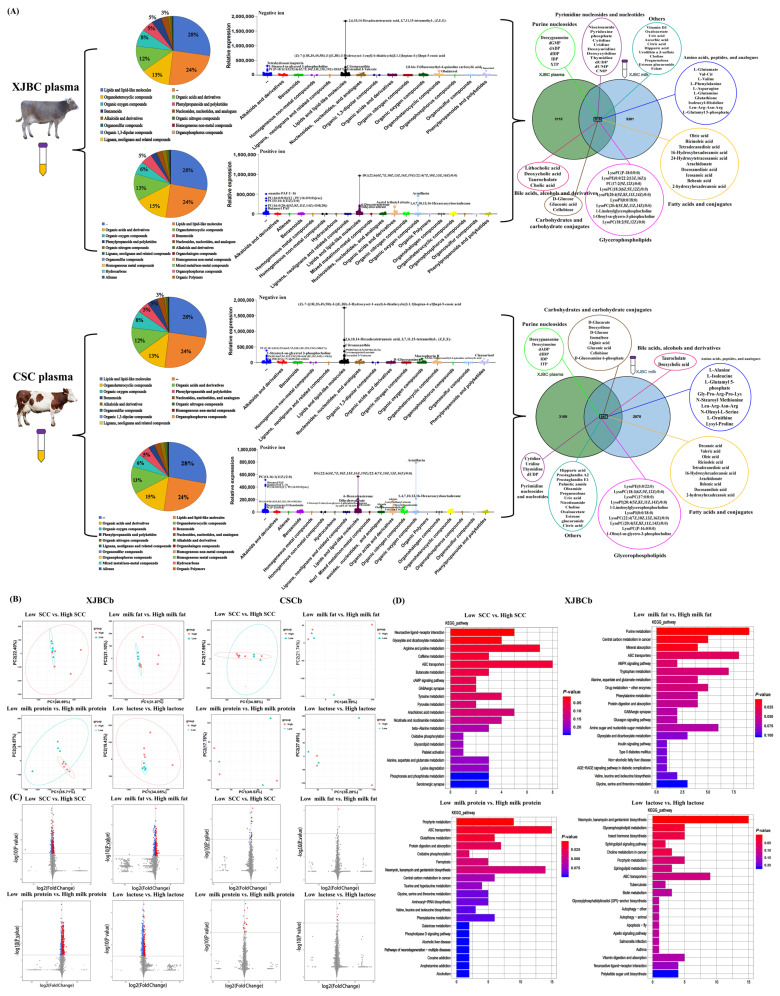
Metabolomics reveals the association between plasma metabolite expression and milk DHI indicators. (**A**) Expression patterns of metabolites in plasma of XJBC and CSC. Pie charts of metabolite classification in XJBC plasma and CSC plasma. In the scatter plot, we added a median bar with a range for each class of metabolites, and each point represents one type of metabolite. We annotated the names of metabolites above the partial points. The Venn diagram showed the number of common metabolites in plasma and milk; (**B**) PCA of metabolome samples. (**C**) Volcanic map of DEGs. Number (*n*) of samples in each group of XJBC: low or high SCC group: *n* = 5; low or high milk fat percentage group: *n* = 5; low or high milk protein percentage group: *n* = 5; and low or high lactose percentage group: *n* = 5. Number (*n*) of samples in each group of CSC: low or high SCC group: *n* = 5; low or high milk fat percentage group: *n* = 3; low or high milk protein percentage group: *n* = 3; low or high lactose percentage group: *n* = 3; (**D**) KEGG enrichment analysis of DEMs.

A total of 4026 metabolites were detected in XJBC plasma. In total, 1903 metabolites were detected under the negative ion mode, of which 524 were lipids and lipid-like molecules. Moreover, 234 were organic acids and derivatives, 247 were organo-heterocyclic compounds, 153 were organic oxygen compounds, and 100 were phenylpropanoids and polyketides. In total, 2123 metabolites were detected in the positive ion mode, of which 507 were lipids and lipid-like molecules, 313 were organic acids and derivatives, 272 were organo-heterocyclic compounds, 129 were organic oxygen compounds, and 105 were benzenoids (Figure 3A).

A total of 4016 metabolites were detected in CSC plasma. In total, 1895 metabolites were detected in the negative ion mode, of which 524 were lipids and lipid-like molecules, 247 were organo-heterocyclic compounds, 234 were organic acids and derivatives, and 153 were organic oxygen compounds. Moreover, 2121 metabolites were detected in the positive ion mode, of which 507 were lipids and lipid-like molecules, 313 were organic acids and derivatives, 272 were organo-heterocyclic compounds, 129 were organic oxygen compounds, and 108 were benzenoids (Figure 3A).

The basic composition of plasma metabolites was similar to that of milk metabolites (milk metabolome data came from our previous studies). Lipids and lipid-like molecules were highly expressed in both plasma and milk. In addition, it is worth noting that plasma contains many basic metabolites related to animal tissue/cell development, sugar, protein, and lipid biosynthesis, of which 920 metabolites are shared between milk metabolites and plasma metabolites (Figure 3A).

### 2.4. DEM Analysis

The PCA of metabolome samples showed (Figure 3C) that samples with high phenotypic value and samples with low phenotypic value were not significantly distinguished in different comparison groups. In XJBC, samples with low phenotypic values were included within the range of samples with high phenotypic values.

DEM and KEGG enrichment analyses (Figure 3C,D, Supplementary Files S4 and S6) showed that 317 DEMs (148 downregulated and 169 upregulated) were identified from the low SCC vs. high SCC in XJBC. These DEMs were involved in pyrimidine metabolism, purine metabolism, arginine and proline metabolism, ABC transporters, etc. Among them, gamma-aminobutyric acid, acetoacetyl-CoA, L-homoserine, succicnic acid, pyrroglutamic acid, glycerol 3-phosphate, uridine 5′-diphosphate, tyamine, L-dopa, deoxycytidine, and uridine were key nodes in the DEM–DEM interaction network (Figure S2). Next, 281 DEMs (77 downregulated and 204 upregulated) were identified in the low milk fat percentage vs. high milk fat percentage group, and they were also involved in ABC transporters, purine metabolism, etc. Among them, 5-hydroxyindoleacetic acid, L-phenylalanine, choline, L-glutamine, mannitol 1-phosphate, deoxycytidine, guanosine, and D-glucose were key nodes in the DEM–DEM interaction network (Figure S2). In addition, 597 DEMs (340 downregulated and 257 upregulated) were identified from the low milk protein percentage vs. high milk protein percentage group. DEMs were enriched relative to arginine and proline metabolism, ABC transporters, phenylalanine metabolism, cysteine and methionine metabolism, glutathione metabolism, amino sugar and nucleotide sugar metabolism, protein digestion and absorption, tryptophan metabolism, etc. Among them, NADP, glutathione, succinic acid, and L-glutamic acid were located in the key nodes of the DEM–DEM interaction network (Figure S2). Finally, 403 DEMs (242 downregulated and 161 upregulated) were identified in the low lactose percentage vs. high lactose percentage group. DEMs were involved in vitamin digestion and absorption, steroid hormone biosynthesis, glycerophospholipid metabolism, arginine and proline metabolism, ABC transporters, etc. Among them, uridine 5′-diphosphate, tyramine, deoxyadenosine triphosphate, thymidine, uridine, glycerol 3-phosphate, dimethyl sulfoxide, and succinic acid were key nodes in the DEM–DEM interaction network (Figure S2).

However, very few DEMs have been identified in CSC. In total, 26 (10 downregulated and 16 upregulated), 4 (2 upregulated and 2 downregulated), 19 (6 downregulated and 13 upregulated), and 6 (all upregulated) DEMs were identified in the low SCC vs. high SCC, low milk fat percentage vs. high milk fat percentage, low milk protein percentage vs. high milk protein percentage, and low lactose percentage vs. high lactose percentage groups, respectively.

### 2.5. Joint Analysis of DEGs and DEMs

**Figure 4:**
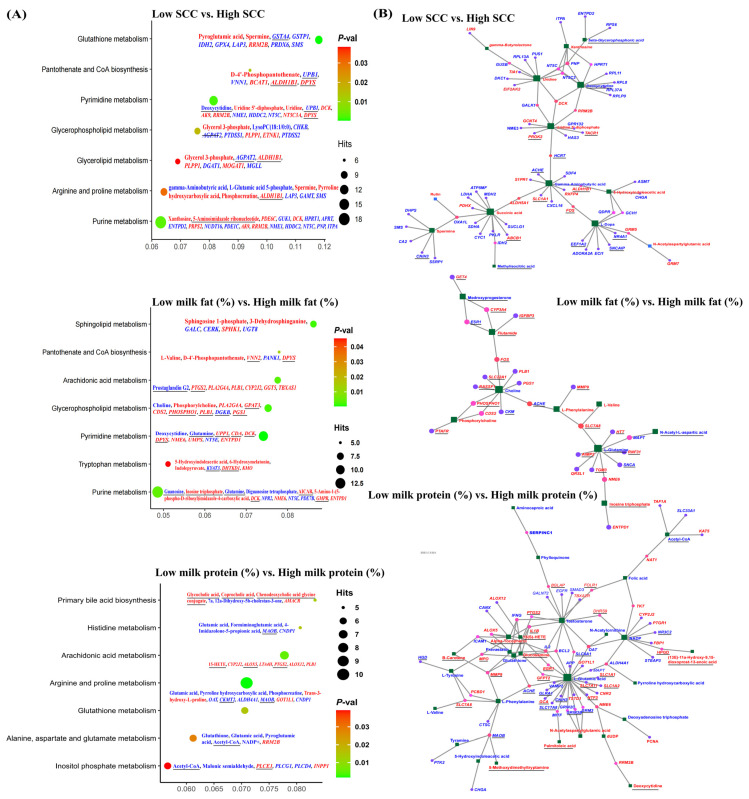
Joint analysis of metabolome and transcriptome. (**A**) KEGG coenrichment analysis of DEGs and DEMs. (**B**) Interaction networks of DEGs and DEMs. In (**A**,**B**), blue DEGs and DEMs were downregulated, and red DEGs and DEMs were upregulated.

KEGG coenrichment analysis showed that (Figure 4A, Supplementary File S5) SCC-related DEGs and DEMs were significantly coenriched relative to glutathione metabolism, pyrimidine metabolism, purine metabolism, pantothenate and CoA biosynthesis, glycerophospholipid metabolism, arginine and proline metabolism, and glycerolipid metabolism. In the interaction network of DEMs and DEGs (Figure 4B), uridine, deoxycytidine, uridine 5′-diphosphate, gamma-aminobutyric acid, succinic acid, spermine, L-dopa, 5-hydroxyindoleacetic acid, *PNP*, *NT5 C*, *NT5 C3*, *GALK1*, *DCK*, *RRM2 B*, *HCRT*, *FOS*, *ALDH5 A1*, and *OXA1 L* genes were located in the key node position. Next, DEGs and DEMs related to milk fat percentage were significantly coenriched relative to pyrimidine metabolism, sphingolipid metabolism, glycerophospholipid metabolism, arachidonic acid metabolism, purine metabolism, pantothenate and CoA biosynthesis, and tryptophan metabolism. Choline, flutamide, L-phenylalanine, L-glutamine, *FOS*, *ACHE*, and *SLC78 A* genes are located in the key nodes of the interaction network. DEGs and DEMs related to milk protein percentage were significantly coenriched relative to arginine and proline metabolism; arachidonic acid metabolism; primary bile acid biosynthesis; glutathione metabolism; histidine metabolism; alanine, aspartate, and glutamate metabolism; and inositol phosphate metabolism. L-glutamic acid, testosterone, NADP, L-phenylalanine, glucosamine, glutathione, pravastatin, alpha-tocopherol, 15(S)-HETE, L-phenylalanine, *IL2*, *BCL2*, *SLC6 A1*, *OAT*, *IL1 B*, *IFNG*, *PTGS2*, *ALOX5*, *ICAM1*, *MPO*, *MMP9*, *ACHE*, *GFPT2*, *EGR1*, and *ALDH4 A1* genes were located at the key nodes of the interaction network. Finally, lactase percentage-related DEGs and DEMs were significantly coenriched relative to glycerophospholipid metabolism and vitamin B6 metabolism.

## 3. Discussion

Blood plays a crucial role in the formation of milk. Blood transports oxygen, nutrients, enzymes, and hormones to bovine mammary tissue and is involved in the response or immune process of bovine mastitis. The above role of blood is inseparable from its two major components: plasma and blood cells (including red blood cells and immune cells: white blood cells, neutrophils, etc.). Plasma is a complex collection of water, protein, fat, inorganic salts, cellulose, and other small-molecule metabolites. It is the medium of material exchange between blood and cells, tissues, and organs, and it is also the metabolic site of many blood cells; thus, its composition is complex and changeable. Based on the joint analysis of blood transcriptome and plasma metabolome, we can systematically understand the influence of blood on SCC, milk fat percentage, milk protein percentage, and lactose percentage.

### 3.1. The Expression of Genes and Metabolites in Blood Affects the SCC

Bovine mammary glands have their own anatomy and immune mechanisms to prevent and overcome infections. The anatomical structure of the papilla is a physical barrier for pathogen entry into the papillary duct, including the papillary skin, papillary sphincter, and keratin plug (which has a bactericidal effect) [25,26]. However, the keratin plug disappears a few days before calving, and the further dilation of milk ducts occurs during milking [27]. As a result, pathogens have the opportunity to enter the nipple tube, and wear and cracks on the skin of the nipple also promote pathogen invasion. When the pathogen enters the milk duct, it will initially destroy the large milk-collecting duct and milk storage pool and produce toxic factors, which further destroy the mammary epithelial cells. These damaged mammary epithelial cells produce attractants that draw immune cells from the blood into the milk [28]. Immune cells have the ability to engulf and destroy pathogens, but if pathogens are not destroyed, they continue to proliferate, increasing the SCC in the milk [29]. The above processes are inseparable from the expression and regulation of genes in immune cells. In addition, as a medium of the exchange and transport of substances, plasma also contains metabolites related to the occurrence and immunity of bovine mastitis, which may be derived from blood immune cells, other tissues, or the diet. They may be directly involved in the development or immune process of mastitis, or they may interact with genes in immune cells and become involved. Compared with dairy cattle, dual-purpose cattle (such as XJBC and CSC) have stronger stress resistance and are good subjects for studying mastitis resistance, but they have received little attention.

In this study, transcriptome data reflected breed specificity (differentiated transcriptome samples based on breed) rather than differentiated transcriptome samples based on the purpose of the cattle. This suggested that the expression patterns of genes in the blood are not related to the purpose of the cattle but rather reflect the physiological state of the individual itself. WGCNA and DEG analysis further revealed that the molecular regulation mechanisms of SCC in XJBC and CSC were similar. Ogorevc et al. summarized the previously identified candidate genes associated with bovine mastitis [30]. Simultaneously, we found that the expression levels of these genes in XJBC and CSC blood were highly similar (Figure S3). This finding also supported the above view.

Meanwhile, the lightyellow and darkred modules were significantly related to SCC, and a large number of genes in the modules were involved in T/B cell proliferation and differentiation, immune and inflammatory responses, interleukin production and regulation, MHC class I/II protein binding, chemotaxis, the NF-κB signaling pathway, ABC transporters, etc. GO and KEGG enrichment analyses of SCC-related DEGs also yielded results that were very similar to those described above. Both T cells and B cells are immune cells in the human body [31,32], while interleukin plays an important role in transmitting information; activating and regulating immune cells; mediating T/B cell activation, proliferation, and differentiation; and inflammation [33]. NF-κB induces the expression of multiple genes through the activation of stimulating factors (virus, tumor necrosis factor, B-cell activating factor, lymphotoxin, etc.) and produces a variety of cytokines involved in inflammatory responses [34]. As a widely existing integrated membrane protein, ABC transporter proteins are closely related to human diseases and drug metabolism, thus attracting much clinical attention [35]. In summary, the SCC or antimastitis process of dual-purpose cattle is regulated by complex molecular mechanisms, involving different types of immune and inflammatory molecular regulatory pathways. It is worth noting that in the lightyellow and darkred modules, some genes related to the above pathways reached significant differential expression levels. Among them, *BOLA-DQB*, *BOLA-DQA2*, *FOS*, *IL10*, and *BOLA-DQA5* genes were common DEGs, and they were significantly differentially expressed in the low SCC vs. high SCC group. *BOLA-DQB*, *BOLA-DQA2*, and *BOLA-DQA5* genes are members of the bovine leukocyte antigen (BOLA) gene family, and they are widely involved in the response and regulation of immune responses [36]. In addition, previous studies have shown that the *IL10* gene is the main regulator of infection immunity [37]. Several members of the *IL* gene family are involved in the regulation of dairy cow mastitis (e.g., *IL6* and *IL8* genes). *FOS* gene family members are conserved in immune tissues and are associated with increased inflammation [38]. These previous reports also provided evidence indicating that they may play a crucial role in regulating SCC or resistance to dual-purpose cattle mastitis. *FOS* and *IL10* genes were also found in Holstein cattle, C0 vs. C40 [23], suggesting that these two genes are universal in the regulation of inflammation resistance in different breeds of cattle.

In the metabolomics analysis, compared with XJBC, we screened fewer metabolites in the low SCC/milk fat percentage/milk protein percentage/lactose percentage vs. high SCC/milk fat percentage/milk protein percentage/lactose percentage of CSC. This may be due to differences in management between the two farms. CSC had better breeding environments and management, resulting in small blood physiological differences between CSC individuals. Therefore, we only conducted a transcriptomic and metabolomics joint analysis in the XJBC population.

Joint analyses further revealed that DEMs and DEGs were significantly enriched in the metabolism of purine, pyrimidine, glutathione, glycerophospholipids, glyceride, arginine, and proline, as well as in the biosynthesis of pantothenic acid and coenzyme A. These metabolic pathways, as well as DEMs and DEGs in these pathways, may be involved in the regulation of SCC or resistance to dual-purpose cattle mastitis. A large number of studies has confirmed that purine and pyrimidine play a unique role in inflammatory responses and are important inflammatory regulators. They can not only affect the proliferation and survival of T cells but also act as the danger signal released during cell lysis, apoptosis, degranulation, or membrane pore formation. Daneshmand et al. [39] showed that the combination of purine and pyrimidine nucleosides had effects on the growth performance, intestinal morphology, digestive enzymes, serum biochemical indexes, and immune function (significantly increasing immune indexes, such as relative weight of bursa of Farnsold and IgA concentration) of broilers. Carver’s research also demonstrated that adding nucleotides to the food can enhance gastrointestinal growth and maturation in animals, and it can also aid the healing of small and large intestine injuries. Humoral and cellular immune indicators are enhanced, and the survival rate after pathogen infection is higher [40].

Glutathione is an important protective antioxidant against oxidative stress in both intracellular and extracellular cells. Recent studies have shown that glutathione plays an important role in immune regulation, extracellular matrix remodeling, apoptosis, and mitochondrial respiration. Rahman [41] and Ghezzi [42] both reported the key role of glutathione in the control of pulmonary proinflammatory processes.

Glyceride is one of the components of blood fat and has roles in storage and transportation. Glycerophospholipids are the most abundant phospholipids in the body. In addition to forming biofilms, glycerophospholipids comprise one of the components of bile and active membrane surface substances, participating in membrane protein recognition and signal transduction. An increasing number of studies have revealed that glycerides and glycerophospholipids play a vital role in the development of illnesses. Wang et al. [43] showed that arachidonic acid metabolism, glycerol metabolism, and glutathione metabolism are key metabolic pathways in the occurrence of upper respiratory tract inflammation induced by particulate matter. Ruiz’s report [44] revealed that changes in the signaling pathways of glycerol ester and glycerophospholipid drive the inflammatory cascade of adrenal myelopathy. Elaine et al. [45] found that postmeal fatty acid and glycerophospholipid responses were linked to fasting inflammation in Guatemalan adults. A study carried out by Shen et al. further found that lipids may contribute to the diagnosis of mastitis, the development of prevention strategies, and early treatment interventions to improve the health of dairy cows [46]. In addition, Wang et al. [47] showed that glutathione metabolism, purine metabolism, glycerophospholipid metabolism, and biosynthesis of pantothenic acid and coenzyme A may be involved in inflammation.

Arginine and proline are two important amino acids, both of which are involved in a variety of important metabolic processes, and their roles in the regulation of bovine mastitis should not be ignored. Arginine comprises a variety of biological protein components, including stomach proteins, antibodies, immunoglobulins, hormones, milk secretions, etc. It is also used as a raw material for antibiotics. Asthma is a complex chronic inflammatory lung disease. King [48] recently found that arginine metabolism is involved in the coping process of asthma. Satriano [49] found that arginine can respond to injuries caused by acute inflammation, such as exhibiting an early response to wound healing or experimental glomerular inflammation. It is worth noting that there is an interaction relationship between the *FOS* gene and gamma-aminobutyric acid, which is a DEM in arginine and proline metabolic pathways. This may also indicate that the *FOS* gene regulates SCC, or mastitis resistance, by influencing arginine metabolism. In summary, the above results provide a new idea for follow-up research on the regulatory mechanism of mastitis resistance.

### 3.2. Blood Metabolism Affects Milk Fat Percentage, Milk Protein Percentage, and Lactose Percentage

The regulation of blood on SCC is obvious; we also found that SCC is positively correlated to milk fat and milk protein percentages in XJBC or CSC, and it is negatively correlated with lactose percentage. According to the report of Sharma et al. [50], during mastitis infection, damaged bovine mammary epithelial cells may result in a decrease in the content of some components in milk and milk yields, and pathological changes in mammary tissue may also result in leakage and, thus, increase in some components in milk. Both Sharma et al. [50] and Pyorala et al. [51] reported that the content of lactose in milk from cows with mastitis was reduced. Ma et al. reported that the ratio of protein to fat in mastitis milk was higher than that in normal milk, and the increase in milk fat percentage and milk protein percentage may also be related to the decrease in milk production (reducing the dilution effect of milk) [52]. These findings are similar to our own.

We further found that DEGs related to milk fat percentage, milk protein percentage, and lactose percentage were also significantly enriched in immune or inflammatory response-related pathways. Simultaneously, WGCNA showed that gene expression patterns in the lightyellow module significantly affected both SCC and milk fat percentage (the module contained genes associated with inflammation and immunity). These results indicate that SCC shares a common molecular regulatory basis with milk fat percentage, milk protein percentage, and lactose percentage. We hypothesized that DEGs may affect milk fat percentage, milk protein percentage, and lactose percentage through related immune or inflammatory response pathways.

In addition, WGCNA also showed that the darkgreen and gray modules were only related to milk fat percentage, and genes in the darkgreen and gray modules were involved in lipid metabolism. This suggested that gene expression patterns in the blood may affect milk fat percentages. Joint analyses further revealed that the DEMs and DEGs associated with milk fat percentage were significantly coenriched relative to more types of lipid metabolic pathways (including glycerophospholipids, sphingolipids, and arachidonic acid metabolism). Arachidonic acid is an essential fatty acid in animals that plays an important role as a structural phospholipid-binding lipid in blood, liver, muscle, and other organ systems. Long-chain polyunsaturated fatty acids such as arachidonic acid are thought to be essential for early neonatal brain and retinal development. Sphingolipids and their metabolites are important active molecules involved in the regulation of cell growth, differentiation, aging, and programmed cell death. Both glycerophospholipids and sphingolipids are also composed of fatty acids. Milk fat is composed of phospholipids, cholesterol, and triglycerides, and the biosynthesis of milk fat occurs in mammary epithelial cells. Fatty acids, including arachidonic acid, are important substrates for milk fat biosynthesis [53], which are obtained from food digestion and transported to breast epithelial cells through the blood. We considered that lipid metabolism in blood may affect milk fat biosynthesis. DEGs and DEMs, which were enriched relative to the abovementioned lipid metabolic pathways, may be involved in this regulatory process. In addition, among the 142 fatty acids and their detected conjugates, 92 in the high milk fat percentage group exhibited lower expression levels than those in the low milk fat percentage group (Figure S4). Based on the above findings, we considered that the reasons for the above results were as follows: (1) The fatty acids in blood and their conjugates are transferred to the breast tissue in large quantities for milk fat synthesis or eventually into milk. Based on our previous milk metabolomics study, out of 45 common fatty acids and their conjugates, 30 are expressed more in milk than in plasma (Figure S5). (2) Milk fat percentages were significantly correlated with SCC, and pathological changes in the mammary epithelial tissue may also result in fatty acid leakage from the blood into milk. (3) Increased immune cell activity and lipid metabolism resulted in the increased consumption of fatty acids and their conjugates in the blood. We also found that there was an interaction between the *FOS* gene and choline, a DEM related to milk fat percentage, and choline was significantly enriched in the metabolism pathway of glycerophospholipid, suggesting that the *FOS* gene might be involved in the regulation of phospholipid metabolism and, thus, affect the milk fat percentage. Haubert et al. found that treatment with choline and the FOS protein resulted in a reduction in total mean fat in the liver and heart tissue [54]. The result supports our conclusion.

Milk proteins are mainly composed of casein, α-lactalbumin, and β-lactoglobulin [25]. They are biosynthesized in mammary epithelial cells by amino acids that are broken down via the digestion of food. However, amino acids also need to be transported through the blood to the mammary epithelial cells for milk protein biosynthesis. In our study, we found that the expression levels of 24 amino acids or short peptides of the high milk protein percentage group were lower than those of the low milk protein percentage group, indicating that there were certain differences in the metabolism levels of amino acids or short peptides between the two groups. This finding was further supported via the KEGG coenrichment analysis, which showed that the DEGs and DEMs associated with the milk protein rate were coenriched in more types of amino acid metabolic pathways (including glycine, serine, threonine, arginine, proline, histidine, alanine, aspartic acid, glutamic acid, tryptophan, tyrosine metabolism, and lysine degradation). Therefore, we also speculated that amino acid metabolism in blood may affect milk protein biosynthesis. DEGs and DEMs, which were enriched relative to the abovementioned amino acid metabolic pathways, may be involved in this regulatory process. In addition, our results cannot rule out the effect of inflammation or increasing SCC on amino acid metabolism in the blood. Zhang et al. found that lysine is a diagnostic marker of subclinical mastitis using LC-MS/MS [55]. Dervishi et al. found that serine and proline could be used for the diagnosis of early subclinical mastitis during the lactation stages of 4 to 8 weeks postpartum [15]. Haxhiaj et al. also found that valine, serine, tyrosine, and phenylalanine can be used in the diagnosis of subclinical mastitis [17].

## 4. Conclusions

The expression pattern of genes in blood mainly affects the SCC and milk fat percentage. In blood, the signaling pathways related to immune or inflammatory responses play a key role in the regulation of SCC, milk fat percentage, milk protein percentage, and lactose percentage. The metabolism of purine, glutathione, glycerophospholipid, glycine, arginine, and proline in blood was also related to SCC, while the metabolism pathways of lipids and amino acids in blood were related to milk fat percentage and lactose percentage, respectively. DEGs and DEMs located in the above pathways may be involved in the regulation of SCC, milk fat percentage, milk protein percentage, and lactose percentage.

## 5. Materials and Methods

### 5.1. Sample Collection

In this study, XJBC and CSC cattle were obtained from the Yili Xinhe cattle breeding farm (Yili, China) and Chuangjin Benniu Cattle Co., Ltd. (Yili, China), respectively. We conducted a population survey of lactating XJBC and CSC herds and collected their basic information: birth date, parity, and lactation stage. These cattle were raised in an environment with suitable temperature and humidity levels, and they were fed a total mixed-ration diet. Experienced feeding managers collected milk and blood during feeding. We collected 50 mL of milk from each cow and placed it in a 50 mL centrifuge tube for the DHI assay. Subsequently, all milk samples were assayed for DHI using the FOSS CombiFoss 7 (FOSS, Denmark). The Pearson correlation between the SCC, milk fat percentage, milk protein percentage, and lactose percentage was analyzed using SPSS 25.

Next, based on the DHI phenotype and basic information of XJBC and CSC, cattle (all cattle were similar in age (from 3 to 3.5 years old) and parity (parity of 1 or 2) and were in the postlactation stage) with a high SCC (XJBC: number of plasma samples/number of buffy coat samples = 5/5; CSC: number of plasma samples/number of buffy coat samples = 5/5) (or high milk fat percentage (5/5; 3/5), high lactose percentage (5/5; 3/5) and milk protein percentage (5/5; 3/5)), and low SCC (XJBC: number of plasma samples/number of buffy coat samples = 5/5; CSC: number of plasma samples/number of buffy coat samples = 5/5) (or high milk fat percentage (5/5; 3/5), high lactose percentage (5/5; 3/5), and milk protein percentage (5/5; 3/5)) were selected for the collection of peripheral blood. In total, 10 mL of peripheral blood was taken from the tail of the cow using a vacuum sampling vessel, which was then placed in a centrifuge at 3500 rpm and centrifuged for 15 min. After the plasma samples were aspirated with a Pasteur pipet and placed in a 1.5 mL Ep tube, they were immediately transferred to a −80 °C refrigerator for storage. Then, the buffy coat was absorbed into a new 1.5 mL Ep tube with a pipette, 1 mL of Trizol reagent was added, and the buffy coat was fully lysed via vortexing and shaking for 3 min. After lysis, the buffy coat samples were stored in a refrigerator at −80 °C for total RNA extraction. During the entire experiment, we strictly followed the policies of the Animal Welfare Committee of Xinjiang Agricultural University.

### 5.2. Metabolites and Total RNA Extraction

Total RNA was extracted from buffy coat samples using Trizol reagent (Invitrogen, Carlsbad, CA, USA). The extraction was carried out according to the kit’s instructions. After extraction, the OD 260/280 and OD 260/230 of total RNA were determined using NanoDrop 2000 (Thermo Fisher, Waltham, MA, USA), and the total RNA integrity (RIN) was determined using Agilent 2100 (Agilent, Santa Clara, CA, USA). All RNA samples had OD 260/280 values within the range of 1.8~2.0, OD 260/230 values greater than 2.0, and RIN values within the range of 7.0~8.5, which could be used for library construction.

The extraction of metabolites in plasma samples was processed [56] as follows: (1) 100 μL of the plasma sample was used, and 500 μL of an extraction solution containing the internal standard was added (the volume ratio of methanol to acetonitrile was 1:1; the internal standard’s concentration was 20 mg/L), swirled, and mixed for 30 s; (2) the mixed sample was placed in the ultrasonic machine and shaken for 10 min (ice water bath); (3) the sample was placed in a −20 °C environment for one hour; (4) the sample was then centrifuged at 4 °C at 13,400 G for 15 min; (5) 500 μL of the supernatant was taken and placed in an EP tube; (6) the extracts were dried using a vacuum concentrator; (7) 160 μL of the extraction solution (volume ratio of acetonitrile to water 1:1) was added to the dried metabolites for redissolution; (8) after redissolution, the sample was vortexed for 30 s and then placed in the ultrasonic machine for 10 min (ice water bath); (9) the samples were centrifuged at 13,400 G for 15 min at 4 °C; (10) 120 μL of the supernatant was taken from a 2 mL sample bottle for LC-MS/MS analysis; and 10 μL of each sample was taken and mixed into the QC sample for quality control.

### 5.3. Sequencing Library Construction and RNA-Seq

Library construction and RNA-Seq were carried out by BGI Gene Technology Service Co., Ltd. (Shenzhen, China) after the total RNA was qualified. Library construction was carried out with reference to the instructions of the sequencing platform. (1) A certain amount of total RNA samples was taken, and the mRNA was obtained from the total RNA using oligo dT. (2) The mRNA was then separated into fragments. (3) The first strand of cDNA was synthesized using the fragmented mRNA as a template and the addition of random primers. (4) The second cDNA strand was then synthesized. (5) Afterward, the double-stranded cDNA was end-repaired, an “A” tail was added, and the sequencing connector was connected. (6) The connected products were further subjected to PCR reaction, and the PCR products were recovered. (7) The library products were looped after the library passed quality control. (8) Circular DNA molecules were replicated by rolling rings to form DNA nanospheres (DNBs). (9) RNA-Seq was performed using the DNB-SEQ platform, and the sequencing length was PE150.

### 5.4. LC-MS/MS Analysis

The LC/MS system for metabolomics analysis was carried out using a Waters Acquity I-Class PLUS ultra-high-performance liquid tandem Waters Xevo G2-XS QT high-resolution mass spectrometer [57,58]. The column used was purchased from Waters Acquity UPLC HSS T3 column (1.8 μm 2.1*100 mm).

The positive ion mode comprised the following: mobile phase A: 0.1% formic acid aqueous solution; mobile phase B: 0.1% formic acid acetonitrile.

The negative ion mode comprised the following: mobile phase A: 0.1% formic acid aqueous solution; mobile phase B: 0.1% formic acid acetonitrile; injection volume: 1 μL.

The Waters Xevo G2-XS QTOF high-resolution mass spectrometer collected primary and secondary mass spectrometry data in the MSe mode via acquisition software (MassLynx V4.2, Waters, Milford, MA, USA). In each data acquisition cycle, dual-channel data acquisition can be performed relative to both low collision energy and high collision energy simultaneously. The low collision energy was set at 2 V, the high collision energy range was within 10~40 V, and the scanning frequency was 0.2 s for mass spectrum examination. The parameters of the ESI ion source were as follows: capillary voltage: 2000 V (positive ion mode) or −1500 V (negative ion mode); cone voltage: 30 V; ion source temperature: 150 °C; desolvent gas temperature 500 °C; backflush gas flow rate: 50 L/h; desolventizing gas flow rate: 800 L/h.

### 5.5. Transcriptome Data Preprocessing

The raw data of RNA-Seq were filtered to obtain clean data for further analysis. Data filtering was performed using FastQ 0.12.1 software [59], and the filtering conditions were as follows: (1) reads matching 25.0% or more of the adapter sequence (allowing a maximum of 2 base mismatches) were deleted; (2) reads less than 150 bp in length were deleted; (3) reads with an *n* ratio ≥ 0.1% were deleted; (4) reads with a polyX value (X can be A, T, G, or C) longer than 50 bp were deleted; (5) reads were deleted if bases with mass values below 20 accounted for 40.0% or more of reads. After the quality control phase, the number of clean reads in all transcriptome samples ranged from 1201 × 10^6^ to 1204 × 10^6^, Q20 ranged from 97% to 98%, Q30 ranged from 93% to 94%, and the GC content ranged from 49% to 52%.

Clean data were mapped to the reference genome (ARS-UCD 1.3, GCF_002263795.2) of the cattle (*Bos taurus*) using Hisat 2.0 software [60]. The mapping rate of all transcriptome samples was greater than 94%. Then, StringTie 2.2.0 software was used to assemble and quantify transcripts in order to obtain the count value of the entire genome’s genes, which was then normalized to the CPM (counts per million) value [61].

### 5.6. Metabolome Data Preprocessing

The raw data were collected via MassLynx V4.2 and further processed using Progenesis QI software. Then, metabolite identification was performed using Progenesis QI [62] based on the METLIN database (http://metlin.scripps.edu (accessed on 15 March 2024)), public databases (including KEGG (https://www.genome.jp/kegg/pathway.html (accessed on 15 March 2024)), HMDB (https://hmdb.ca/ (accessed on 15 March 2024)), Lipidmaps (https://lipidmaps.org/databases (accessed on 15 March 2024))), and the self-built database of BioMarker Technologies Co., Ltd. (Beijing, China). Finally, theoretical fragment identification was carried out. The deviation of the parent ion mass number was 100 ppm, and the deviation of the fragment ion mass number was less than 50 ppm.

### 5.7. Bioinformatic Analysis

#### 5.7.1. Bioinformatics Analysis of Transcriptome Data

The principal component analysis (PCA) of transcriptome data was performed using the OECloud tool (https://cloud.oebiotech.com (accessed on 24 June 2024)). Then, whole genome gene count values of all transcriptome samples were used as input data for weighted gene coexpression network analysis using WGCNA 1.72 [62]. In WGCNA, a cluster tree was first drawn according to the correlation between samples, and a pairing Pearson correlation matrix between all genes was constructed. To achieve scale-free networks, the pick Soft Threshold function was used to calculate the adjacency between genes with an appropriate soft threshold (β). Finally, in order to identify gene modules, the topological overlap measure (TOM) was used to calculate correlation [63], and a hierarchical clustering tree was constructed according to dissimilarity (1-TOM) with a minimum module size of 30 [64]. Genes with similar expression patterns were grouped into the same module. Modules with greater than 75% similarity were merged using the default 0.25 tree height cut in WGCNA [65,66]. The statistical significance of the correlation between the module and the SCC, milk fat percentage, milk protein percentage, and lactose percentage was verified via the Pearson correlation, and a module with *p* < 0.05 was the most critical [67]. DESeq 2.0 was used to conduct DEG analyses between the groups with high and low SCC/milk fat percentage/milk protein percentage/lactose percentage [68]. In the DEG analysis, |log2(Fold Change)| ≥ 1 and *p* < 0.05 (*p* values were adjusted using the Benjamini and Hochberg approach to control the false discovery rate) were used as the screening thresholds for DEGs.

Gene ontology (GO) annotation and the Kyoto Encyclopedia of Genes and Genomes (KEGG) pathway enrichment analysis of DEGs were performed using DAVID online software (https://david.ncifcrf.gov/ (accessed on 25 June 2024)). GO annotations are classified by function into biological processes (BPs), cellular components (CCs), and molecular functions (MFs). When *p* < 0.05, the term or pathway was considered to be significantly enriched. Finally, the protein–protein interaction (PPI) network for DEGs was constructed using the online STRING website (https://cn.string-db.org/ (accessed on 25 June 2024)).

#### 5.7.2. Bioinformatics Analysis of Metabolome Data

The PCA of metabolome data was performed using the prcomp 3.6.1 software (scale method: uv scaling). In DEM analysis, the ropls 1.6.2 software was used to perform the orthogonal partial least squares discriminant analysis (OPLS-DA) (cross-verification fold number: 7 (if there were fewer than seven samples, the fold number = sample number); the number of replacement tests is 200) [69]. R^2^ Y and Q^2^Y were used to assess the predictive ability of the model (whether the model could distinguish the correct sample groups relative to the amount of metabolite expression). The model’s variable importance in projection (VIP) value was calculated using multiple cross-validations. MetaboAnalyst R 6.0 [70] and pROC 1.15.0 [71] were used to analyze receiver operating characteristic (ROC) curves, and the AUC (area under the curve) value for each metabolite was determined. Based on the *p*, VIP, and AUC values were adopted to screen the DEMs. The screening thresholds were *p* < 0.05, VIP > 1, and AUC > 0.75. Finally, the KEGG database was used for the functional enrichment analysis of DEMs, and MetaboAnalyst 6.0 software (https://www.metaboanalyst.ca/MetaboAnalyst/home.xhtml (accessed on 26 July 2024)) was used to conduct the DEM–DEM interaction network analysis.

#### 5.7.3. Joint Analysis of Metabolome and Transcriptome Data

The network function of the MetaboAnalyst 6.0 online website was used for the KEGG coenrichment analysis of DEGs and DEMs, as well as the interaction network analysis of DEGs and DEMs, and the parameters were set at the default settings. During the joint analysis, we lowered the screening threshold for DEGs (*p* < 0.05 as the screening threshold for DEGs) to keep the screening thresholds of DEGs relatively consistent with those of DEMs, thereby obtaining more comprehensive KEGG coenrichment results and DEGs–DEMs interaction networks.

## Figures and Tables

**Table 1:** Correlation analysis of SCC, milk fat percentage, milk protein percentage, and lactose percentage.

Breed		SCC	Milk Fat	Milk Protein	Lactose
XJBC	SCC	1	0.204 **	0.238 **	−0.349 **
	Milk Fat	0.204 **	1	0.115 *	−0.183 **
	Milk Protein	0.238 **	0.115 *	1	−0.433 **
	Lactose	−0.349 **	−0.183 **	−0.433 **	1
CSC	SCC	1	−0.035	0.266 **	−0.391 **
	Milk Fat	−0.035	1	0.147	−0.138
	Milk Protein	0.266 **	0.147	1	−0.0457 **
	Lactose	−0.39 1 **	−0.138	−0.457 **	1

**: *p* < 0.01; *: *p* < 0.05.

## Data Availability

The data and material used in this research are available from the corresponding author upon request.

## Supplementary Materials

The following supporting information can be downloaded at: https://www.mdpi.com/article/10.3390/ijms252212375/s1.
