# Supplementary figures and images for: Transcriptomic and Metabolomics Joint Analyses Reveal the Influence of Gene and Metabolite Expression in Blood on the Lactation Performance of Dual-Purpose Cattle (Bos taurus)
Source: Int J Mol Sci. 2024 Nov 18;25(22):12375. doi: 10.3390/ijms252212375 (PMC11594596; doi:10.3390/ijms252212375)

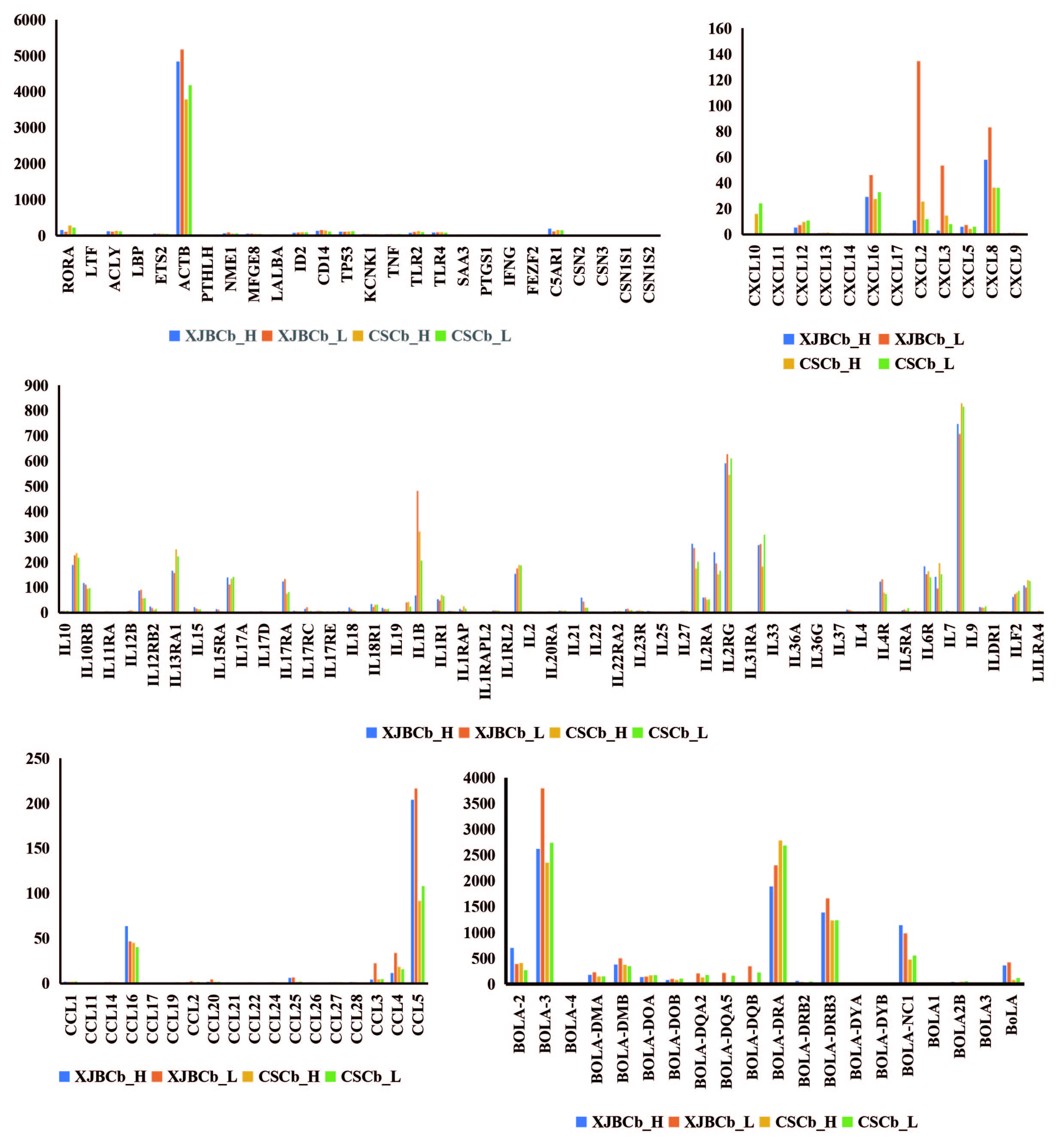

Supplement: Supplementary file 1 [file ijms-25-12375-s001.zip › Figure S3.jpg]

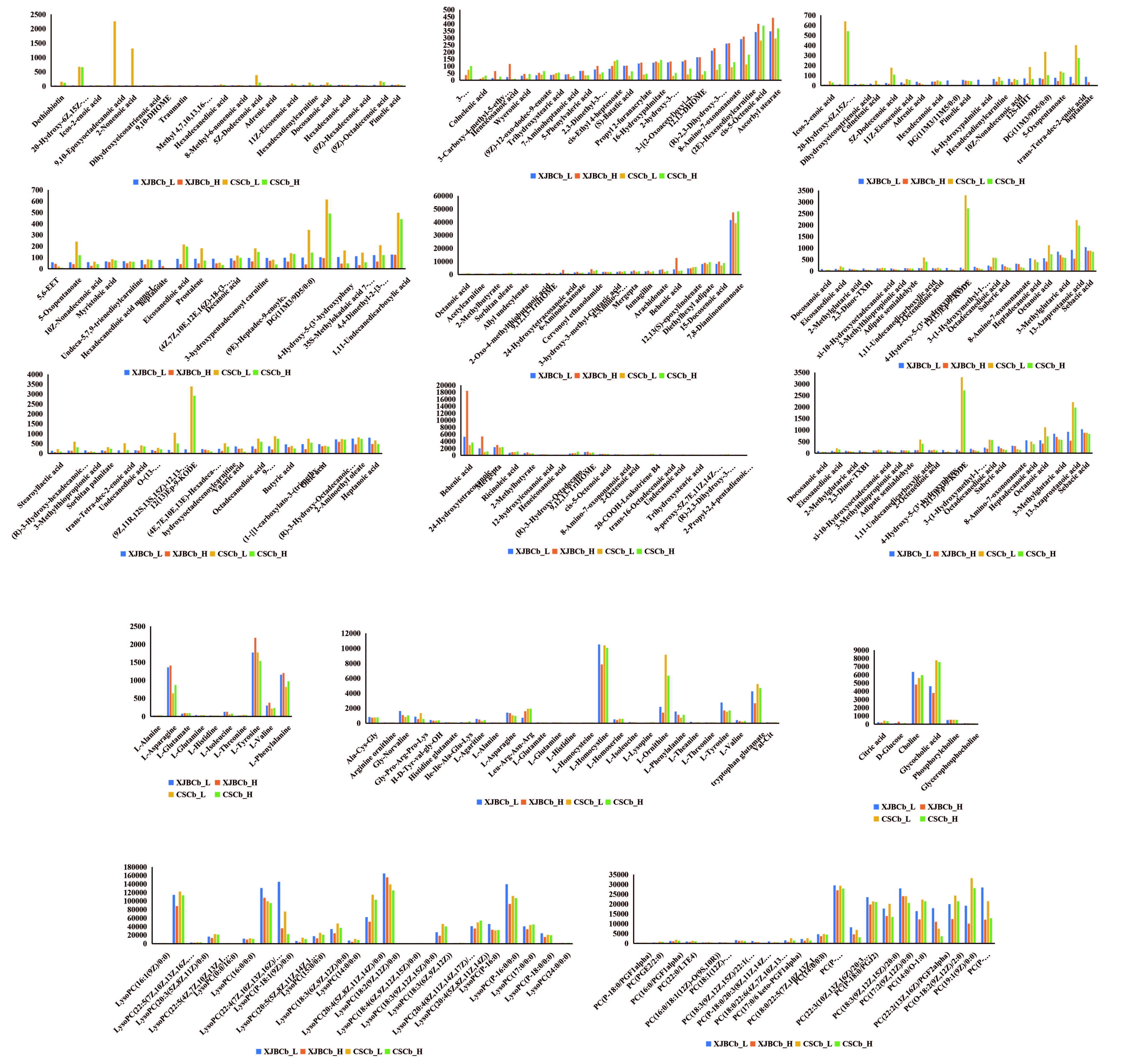

Supplement: Supplementary file 1 [file ijms-25-12375-s001.zip › Figure S4.jpg]

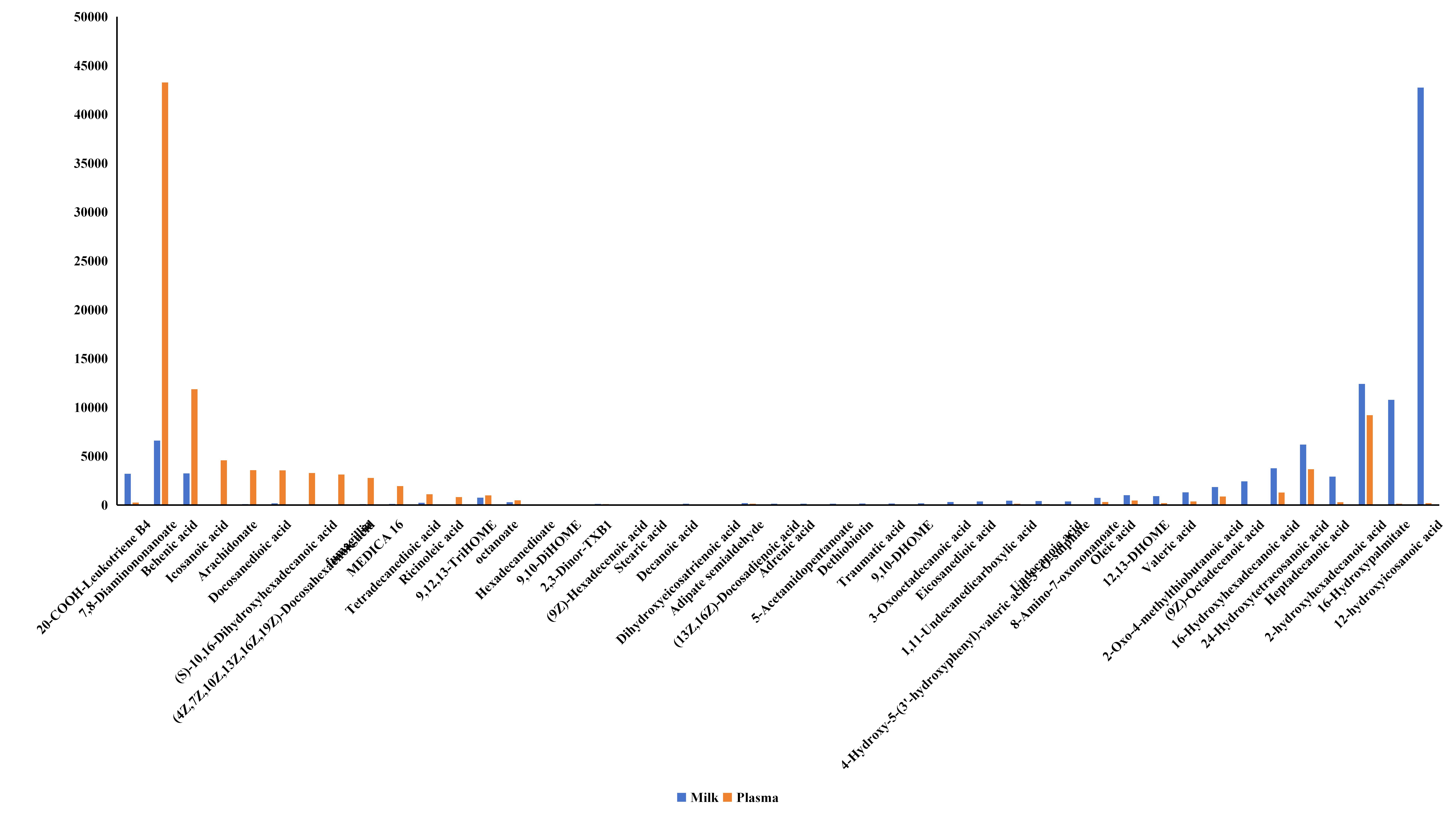

Supplement: Supplementary file 1 [file ijms-25-12375-s001.zip › Figure S5.jpg]
